# Classification of Smoke Contaminated Cabernet Sauvignon Berries and Leaves Based on Chemical Fingerprinting and Machine Learning Algorithms

**DOI:** 10.3390/s20185099

**Published:** 2020-09-07

**Authors:** Vasiliki Summerson, Claudia Gonzalez Viejo, Colleen Szeto, Kerry L. Wilkinson, Damir D. Torrico, Alexis Pang, Roberta De Bei, Sigfredo Fuentes

**Affiliations:** 1 Digital Agriculture, Food, and Wine Group, Faculty of Veterinary and Agricultural Sciences, The University of Melbourne, Parkville, VIC 3010, Australia; vsummerson@student.unimelb.edu.au (V.S.); cgonzalez2@unimelb.edu.au (C.G.V.); alexis.pang@unimelb.edu.au (A.P.); 2 School of Agriculture, Food and Wine, The University of Adelaide, Waite Campus, PMB 1, Glen Osmond, SA 5064, Australia; colleen.szeto@adelaide.edu.au (C.S.); kerry.wilkinson@adelaide.edu.au (K.L.W.); roberta.debei@adelaide.edu.au (R.D.B.); 3 The Australian Research Council Training Centre for Innovative Wine Production, PMB 1, Glen Osmond, SA 5064, Australia; 4 Department of Wine, Food and Molecular Biosciences, Faculty of Agriculture and Life Sciences, Lincoln University, Lincoln 7647, Canterbury, New Zealand; Damir.Torrico@lincoln.ac.nz

**Keywords:** smoke taint, remote sensing, climate change, near-infrared spectroscopy, volatile phenols

## Abstract

Wildfires are an increasing problem worldwide, with their number and intensity predicted to rise due to climate change. When fires occur close to vineyards, this can result in grapevine smoke contamination and, subsequently, the development of smoke taint in wine. Currently, there are no in-field detection systems that growers can use to assess whether their grapevines have been contaminated by smoke. This study evaluated the use of near-infrared (NIR) spectroscopy as a chemical fingerprinting tool, coupled with machine learning, to create a rapid, non-destructive in-field detection system for assessing grapevine smoke contamination. Two artificial neural network models were developed using grapevine leaf spectra (Model 1) and grape spectra (Model 2) as inputs, and smoke treatments as targets. Both models displayed high overall accuracies in classifying the spectral readings according to the smoking treatments (Model 1: 98.00%; Model 2: 97.40%). Ultraviolet to visible spectroscopy was also used to assess the physiological performance and senescence of leaves, and the degree of ripening and anthocyanin content of grapes. The results showed that chemical fingerprinting and machine learning might offer a rapid, in-field detection system for grapevine smoke contamination that will enable growers to make timely decisions following a bushfire event, e.g., avoiding harvest of heavily contaminated grapes for winemaking or assisting with a sample collection of grapes for chemical analysis of smoke taint markers.

## 1. Introduction

The incidence and intensity of wildfires are increasing worldwide, mainly due to the effects of climate change [1,2,3,4,5]. Bushfires that occur near wine regions can result in grapevine smoke exposure, which can alter the chemical composition of grape berries. Wine produced from these smoke-affected grapes may exhibit unpalatable smoky aromas and flavors, such as “burnt wood”, “ashy”, and “burnt rubber” [6,7,8,9]. These undesirable characters have been attributed to smoke-derived volatile phenols (VPs), including guaiacol, 4-methylguaiacol, cresols, and syringol [7,10,11]. It is thought that these VPs accumulate primarily in the skin of grape berries following smoke exposure and, to a lesser extent, in the pulp and seeds [12,13,14,15]. Grapevine smoke exposure, and the resulting smoke taint in wine, have caused significant financial losses for grape growers and winemakers due to discarded grapes and unsaleable wine. For example, the 2009 Black Saturday bushfires in Victoria, Australia, were estimated to have caused AUD 300 million in lost revenue [16,17,18,19]. More recently, the Australian Grape and Wine Incorporated (AWGI) estimated an AUD 40 million loss from the 2019/2020 summer bushfires [20]. Vineyard smoke exposure, therefore, remains a significant issue for the wine industry, particularly given the increasing frequency and severity of bushfires [21].

Grapevine leaves have also been found to accumulate VPs, and a positive correlation has been demonstrated between the levels of smoke compounds detected in leaves and wine when they were included in the primary fermentation [13,22,23]. From a physiological point of view, smoke exposure has also been shown to decrease stomatal conductance in leaves, which may result from the reaction of carbon dioxide (CO_2_) and carbon monoxide (CO) with water vapor in the substomatal cavity producing carbonic acid (H_2_CO_3_) [24,25]. Carbonic acid reduces the pH in the stomata, resulting in partial or complete stomatal closure [25,26]. Damage to leaf surfaces following smoke exposure has also been observed, with the development of necrotic lesions or, in extreme cases, total leaf necrosis [10,22,27]. This may be the result of ozone (O_3_) present in smoke, which has been linked to chlorophyll destruction and accelerated leaf senescence [28,29]. 

Some chromatographic techniques such as gas chromatography-mass spectrometry (GC-MS) and high-performance liquid chromatography-tandem mass spectrometry (HPLC-MS/MS) have been developed to quantify levels of free and glycosidically bound VPs in grapes and wines [30,31,32,33]. While these techniques are currently used for qualitative and quantitative analysis and may assist growers in determining the level of smoke taint in the final wine, there are numerous shortcomings: sample preparation is time-consuming and destructive, and analyses require expensive reagents, standards, and equipment, as well as trained personnel. Furthermore, following a bushfire event, there may be long delays in the availability of results due to large numbers of samples being submitted to commercial laboratories for analysis [34,35]. Consequently, alternative methods of smoke taint analysis have recently been investigated and may offer non-destructive sample preparation, as well as accurate and rapid results.

The use of spectroscopic techniques has increased in recent years due to their ease of use, rapid results, minimal sample preparation, and non-destructive nature, all of which allow repeated measurements to be taken [34,35,36,37,38,39]. Furthermore, the development of smaller, handheld spectroscopic devices coupled with decreasing costs, has allowed these technologies to be more readily accessible and affordable to growers and farmers, while their portability allows for in-field use, reducing the risk of sample deterioration during transportation [39,40]. Ultraviolet (UV) to visible (Vis) spectroscopy involves the region between 200–780 nm, which can be used to analyze compounds containing organic acids, phenolic compounds, and pigments such as anthocyanins, carotenoids, and chlorophylls [41]. UV-Vis spectroscopy has been used to determine the contribution of chemical compounds towards the composition of extra virgin olive oils to determine the region in the Mediterranean it was produced, to optimize the aging process of Spanish wines, and to assess the impact of heating edible oils and to determine their acid level [42,43,44]. Near-infrared (NIR) spectroscopy between the light spectra regions of 780–2500 nm has been widely used in agricultural and food science applications, with NIR bands corresponding to overtones resulting from the vibrations of O-H, C-H, N-H, and S-H bonds [39,41]. Various spectroscopic techniques, most notably in the NIR region, have been used for numerous applications in viticulture, including the assessment of grape quality and ripeness as well as the authentication of geographical origin [38,45,46,47,48,49,50]. Research has also been conducted on the use of mid-infrared (MIR) spectroscopy (between 2500–25,000 nm) of the electromagnetic spectrum, as well as synchronous two-dimensional MIR correlation spectroscopy (2D-COS) for the classification of smoke tainted wines [34,35]. Both techniques showed potential for screening smoke tainted wine, with MIR spectroscopy achieving 61 and 70% classification rates for control and smoke affected wines, respectively. However, classification rates were affected by the degree of smoke taint, as well as compositional differences arising from the grape variety and oak maturation [34]. While this technology may help to assess wine samples for smoke taint, it does not provide an early, in-field detection system that could help growers identify which grapes may be contaminated before winemaking. At present, there is very little research investigating the in-field use of Vis-NIR spectroscopy for the classification of smoke-affected grapevine leaves and berries. Research by Fuentes and coworkers [19] developed a model using NIR spectroscopy between the region of 700–1100 nm to predict the levels of guaiacol glycoconjugates in berries and wine, and the levels of guaiacol in wine. These models may offer growers a non-destructive in-field detection system for grapevine smoke contamination. However, further research is required to determine the effectiveness of different NIR regions for monitoring smoke contamination.

Several chemometric techniques have been used to analyze spectral data, including partial least squares (PLS) regression, principal component analysis (PCA), and artificial neural networks (ANN), to name a few [41]. Of these techniques, ANNs have increased in popularity as classification, prediction, and clustering tools, particularly since they can better interpret the non-linear patterns of spectral data [51,52,53,54]. Machine learning (ML) modeling based on ANN can be trained from a set of given data known as ‘inputs’ or independent variables and form complex, non-linear relationships with these inputs and the ‘targets’ or dependent variables [54]. For example, preliminary ML models for the classification of smoke tainted grapevines have been developed using infra-red (IR) thermal imagery from canopies, which gave an indication of changes in stomatal conductance for classification of control and smoke-exposed grapevines [25]. In addition to this, another model has been proposed that aims to quantify levels of smoke derived compounds in grapes and wine using NIR spectroscopy measurements as inputs [25]. Furthermore, UV-Vis spectroscopy may offer insights into the degree of physiological performance of leaves as well as fruit ripening and quality through analyzing pigment content, such as chlorophylls, anthocyanins, and carotenoids [55,56,57,58,59].

The objective of this study was to investigate the use of NIR spectroscopy, coupled with ML modeling for the detection of grapevine smoke contamination. Grapevine leaves and berries were analyzed in the vineyard in a smoke trial using a NIR spectrometer, and the absorbance values were used as inputs to train different machine learning algorithms in order to create ANNs with the best classification performances. In addition to this, UV-Vis spectroscopy was used to assess the physiological performance and degree of senescence of leaves, as well as the degree of ripening and anthocyanin content of grapes. This may offer growers a rapid and non-destructive detection system that they can employ themselves to obtain real-time information regarding smoke exposure. This will facilitate timely decision-making around which fruit to sample for chemical analysis and/or to harvest to maintain wine quality.

## 2. Materials and Methods

### 2.1. Vineyard Site and Experimental Design for the Smoke Trial

**Figure 1:**
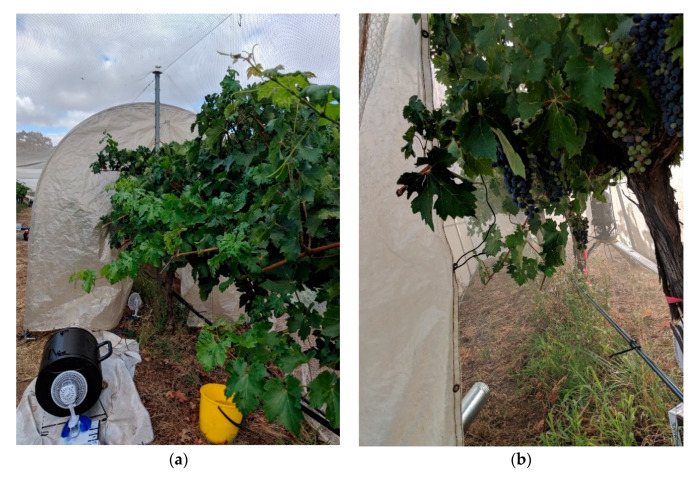
Smoke treatments were applied to grapevines using a purpose-built smoke tent; grapevines were enclosed in the tent and exposed to smoke derived from the combustion of barley straw (**a**,**b**).

The smoke trial was conducted in late January-early February during the 2018/2019 growing season, at the University of Adelaide’s Waite Campus in Urrbrae, South Australia (34°58′ S, 138°38′ E). The trial, described previously by Szeto and colleagues [60], involved the application of smoke and/or in-canopy misting to Cabernet Sauvignon grapevines and comprised five different treatments: a control (C), i.e., neither misting nor smoke exposure; (ii) a control with misting (CM), i.e., in-canopy misting but no smoke exposure; (iii) a high-density smoke treatment (HS); (iv) a high-density smoke treatment with misting (HSM); and (v) a low-density smoke treatment without misting (LS). Treatments were applied to Cabernet Sauvignon grapevines planted in 1998 at 2.0 and 3.3 m vine and row spacings, and trained to a bilateral cordon, vertical shoot positioned trellis system (VSP), hand-pruned to a two-node spur system, with under vine drip irrigation (twice weekly, from fruit set to pre-harvest). Smoke treatments were applied (approximately seven days post-véraison, the period grapes are thought to be most susceptible to smoke contamination [10]) using a purpose-built smoke tent (Figure 1a,b) and experimental conditions reported previously [4,61]: low and high-density smoke treatments were achieved by burning different fuel loads (i.e., ~1.5 and 5 kg of barley straw, respectively). In-canopy misting was evaluated as a method for mitigating the uptake of smoke-derived volatile phenols by grapes and involved the continuous application of fine water droplets (65 µm) to the grapevine bunch zone using a purpose-built sprinkler system (delivering water at 11 L/h), as previously described [62]. Each treatment was applied to six vines from three adjacent panels, except the HS treatment, which comprised only five vines, with treatments separated by at least one buffer vine. LS, HS, and HSM treatments comprised duplicate applications of smoke to 1.5 panels/three vines at a time (except for one HS treatment). The in-canopy sprinkler system was turned on 5 min before the first HSM treatment was applied and off 15 min after the second HSM treatment was completed, such that CM and HSM grapevines were misted for approximately 2.5 h in total. The second and fifth vine from each treatment (the middle vines from smoke treatments) were then selected for physiological and NIR measurements.

### 2.2. Physiological Measurements

The rate of photosynthesis (A), stomatal conductance (g_s_), and transpiration (E) were determined using a portable infrared gas analyzer equipped with a broad leaf chamber (LCpro-SD, ADC Bioscientific Ltd., Hoddesdon, UK). Measurements were taken on three leaves of each side of the canopy per vine (*n* = 12 leaves per treatment) with a photosynthetic photon flux density of 1000 µmol m^−2^ s^−1^ supplied by a high efficiency, low heat output, mixed red-blue light-emitting diode (LED) array unit. Water vapor and CO_2_ concentration in the chamber were set to ambient. Measurements were taken one day (24 h) after smoke treatments were applied, on clear, sunny days.

### 2.3. Determination of Volatile Phenols and Their Glycoconjugates in Grape Juice/Homogenate

The concentration of volatile phenols and their glycoconjugates were determined (in grape juice and homogenate, respectively) using analytical methods described previously [30,32,33,60]. Volatile phenols were measured by stable isotope dilution analysis (SIDA) [3,30,33], using an Agilent 6890 gas chromatograph coupled to a 5973-mass spectrometer (Agilent Technologies, Forest Hill, Vic., Australia). Isotopically labeled standards, i.e., *d*_4_-guaiacol and *d*_3_-syringol, were prepared in-house using methods outlined previously [3,30,33]. The limit of quantitation for volatile phenols was 1–2 µg/L. Volatile phenol glycoconjugates were also measured by SIDA [30,32], using an Agilent 1200 high-performance liquid chromatograph (HPLC) equipped with a 1290 binary pump, coupled to an AB SCIEX Triple Quad^TM^ 4500 tandem mass spectrometer, with a Turbo V^TM^ ion source (Framingham, MA, USA). The preparation of the isotopically labeled internal standard, i.e., *d*_3_-syringol gentiobioside, has been reported previously [30,32]. The limit of quantitation for volatile phenol glycosides was 1 µg/kg.

### 2.4. Near-Infrared Data Collection

Grapevine leaf and berry spectra were collected one day after smoke exposure, using a microPHAZIR^TM^ RX Analyzer (Thermo Fisher Scientific, Waltham, MA, USA), which had a spectral range of 1596 to 2396 nm at intervals of 7–9 nm. Prior to undertaking the measurements and after every 10–15 readings, the device was calibrated using a white background calibration standard (included with the device). The white background was placed on top of the leaf while measuring to avoid signal noise inclusion due to variation in light or environmental changes. Leaves and berries were also analyzed using the Lighting Passport Pro^TM^ handheld spectrometer (Asensetek Incorporation, Xindian District, New Taipei City, Taiwan), which has a spectral range of 380–780 nm at intervals of 1 nm. Measurements were taken at approximately 3 cm from the leaves and berries. All measurements were conducted at ambient temperature between 9:00 a.m. and 6:00 p.m. 

For the leaf spectral measurements, nine sunlit and nine shaded, mature, fully expanded leaves were selected (i.e., 18 leaves per vine, 36 leaves per treatment). Leaves were free of any visible signs of disease or blemishes. Each leaf was measured in three areas, in triplicate, using the microPHAZIR^TM^ RX Analyzer, while three measurements per leaf were taken with the Lighting Passport Pro^TM^ handheld spectrometer. For the berry spectra, two bunches were selected per vine, and nine berries (three from the top, middle, and bottom of each bunch) were measured, in triplicates using the microPHAZIR^TM^ RX Analyzer (n = 540). On the other hand, twelve berries per treatment were analyzed using the Lighting Passport Pro^TM^ (n = 180) while still attached to the bunch. 

### 2.5. Calculating Spectral Indices

Spectral indices for the analysis of pigment content were calculated for both leaves and berries. Leaf spectra taken using the Lighting Passport Pro^TM^ were used to calculate the normalized difference vegetation index (NDVI), normalized anthocyanin index (NAI), plant senescence reflectance index (PSRI), and carotenoid reflectance index (CRI) [56,57,59,63,64,65]. Berry spectra were used to calculate the NAI and PSRI. The calculations and wavelengths used for determining these indices are given in Table 1.

### 2.6. Statistical Analysis

Physiological measurements, spectral indices, volatile phenols, and their glycoconjugates were analyzed by one-way analysis of variance (ANOVA) using Minitab® version 18.1 (Minitab Inc., State College, PA, USA). Mean comparisons were performed using the Fisher least significant difference (LSD) method as a *post-hoc* test at *α* = 0.05. Near-infrared data were analyzed using The Unscrambler X version 10.3 software (CAMO Software, Oslo, Norway). Absorbance values for all wavelengths were plotted for both the microPHAZIR^TM^ RX Analyzer and Lighting Passport Pro^TM^ leaf and berry readings. Principal component analysis (PCA) was also performed using The Unscrambler X program. All microPHAZIR^TM^ RX Analyzer measurements were pre-processed using the second derivative transformation, Savitzky–Golay derivation, and smoothing using The Unscrambler X version 10.3 software prior to the plotting of graphs and statistical analysis.

### 2.7. Artificial Neural Network Modeling 

**Figure 2:**
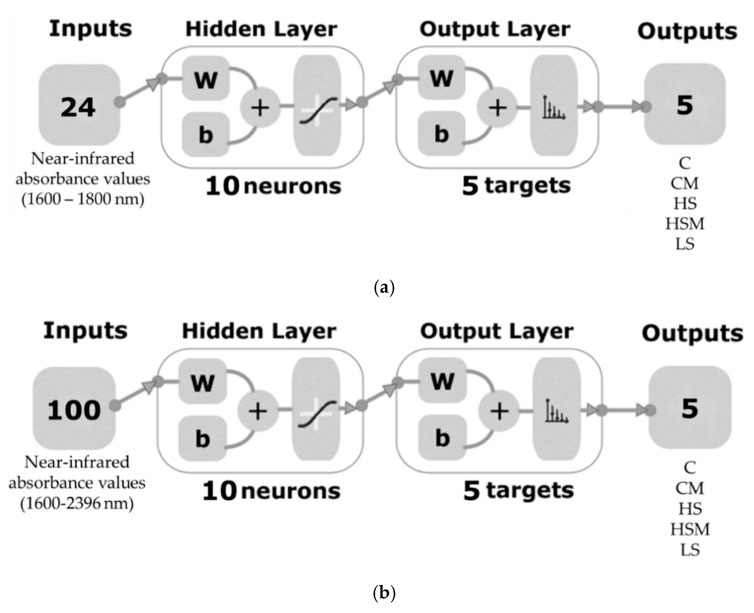
Two-layer feedforward network with ten hidden neurons and sigmoid function for the three classification models: (**a**). microPHAZIR^TM^ leaf model (Model 1) and (**b**). microPHAZIR^TM^ berry model (Model 2). Abbreviations: C = control without misting; CM = control with misting; HS = high density smoke without misting; HSM = high density smoke with misting; and LS = low density smoke.

Three ANN models were developed for berry and leaf NIR readings, which were used as inputs to classify the different smoke treatments using customized code written in MATLAB® (version R2020a, MathWorks Inc., Natick, MA USA) (Figure 2). This code tested a total of 17 training algorithms in a loop to find the optimum in terms of accuracy and performance. Once the optimum training algorithm was identified, further training was performed to develop the most accurate ANN model. For both models, the Levenberg–Marquardt training algorithm was found to be the best algorithm, resulting in models with the highest accuracy and no signs of overfitting. 

Overtones within the 1596–1800 nm range were used as inputs for the microPHAZIR^TM^ leaf model (Model 1). This region was selected to avoid water overtones and any classification resulting from the water status of the vines. The entire spectral range was used for the microPHAZIR^TM^ berry model (Model 2) (1596–2396 nm). The two models were developed using a random data division with 70% (*n* = 1134 for Model 1 and 378 for Model 2) training, 15% (*n* = 243 for Model 1 and 81 for Model 2) for validation with a mean squared error (MSE) performance algorithm and 15% (n = 243 for Model 1 and 81 for Model 2) for testing with a default derivative function. Ten hidden neurons were selected for each of the two models after conducting a trimming exercise with three, five, and ten neurons. 

## 3. Results

### 3.1. Physiological Measurements

Results of gas exchange parameters are shown in Table 2. The transpiration rate was lower for the HS treatment (*P* < 0.005) with a mean rate of 1.43 mmol m^−2^ s^−1^, while no differences were observed in the other treatments. The CM and C treatments both had the highest g_s_ values with an average value of 0.15 mol m^−2^ s^−1^ for each, while HS and LS treatments had the lowest average g_s_ at 0.056 mol m^−2^ s^−1^ and 0.082 mol m^−2^ s^−1^ respectively. Mean rates of A were found to be highest in the C and CM treatments (10.77 µmol m^−2^ s^−1^ and 9.66 µmol m^−2^ s^−1^, respectively), while the LS and HS treatments had the lowest (7.01 µmol m^−2^ s^−1^ and 5.59 µmol m^−2^ s^−1^, respectively).

### 3.2. Levels of Smoke Taint Marker Compounds in Grape Juice/Homogenate

Differences in volatile phenol concentrations between HS and HSM treatments were found for guaiacol, 4-methylsyringol, and syringol (*P* < 0.05; Table S1). In particular, 4-methylsyringol and syringol had the largest differences in concentrations amongst the smoke treatments, with the HS treatment exhibiting the highest mean values (17 and 126 µg/L, respectively) followed by the HSM treatment (9 and 59 µg/L, respectively) while the CM treatments exhibited the lowest mean values (2 and 8 µg/L), which displayed the lowest mean value. There were no differences between the HS and HSM treatments, nor between the C, CM, and LS treatments for 4-methylguaiacol, phenol, and total cresols; however, HS and HSM grapes had significantly higher volatile phenol concentrations than C, CM, and LS grapes. 

Some differences in volatile phenol glycoconjugate levels could be seen amongst the five smoke treatments. Some glycoconjugates displayed differences between the HS and HSM treatments. There was no difference in GuRG levels between the LS, HS, and HSM treatments, with no levels detected in the C and CM treatments. The HS smoke treatment had the highest levels of PhRG, PhGG, CrPG, SyGG, and SyPG, followed by the HSM and LS treatments and then the C and CM treatments. Interestingly the C and HS treatments had the highest level of CrGG followed by the CM and HSM treatment, while the LS treatment had the lowest concentration. 

### 3.3. NIR Absorbance Patterns for Leaves and Berries

**Figure 3:**
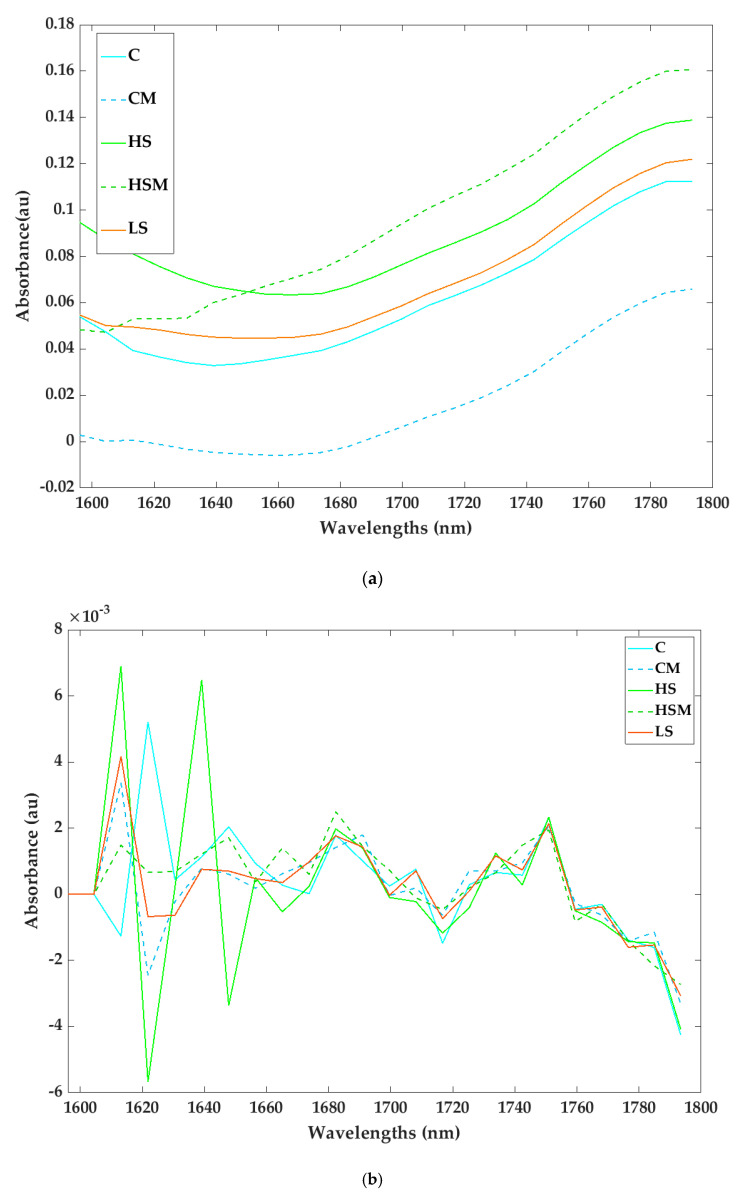
Raw leaf absorbance (**a**) and second derivative spectra (**b**) measured with the microPHAZIR^TM^ near-infrared (NIR) analyzer for the different smoke and misting treatments. Abbreviations: C = control without misting; CM = control with misting; HS = high density smoke without misting; HSM = high density smoke with misting; and LS = low density smoke.

**Figure 4:**
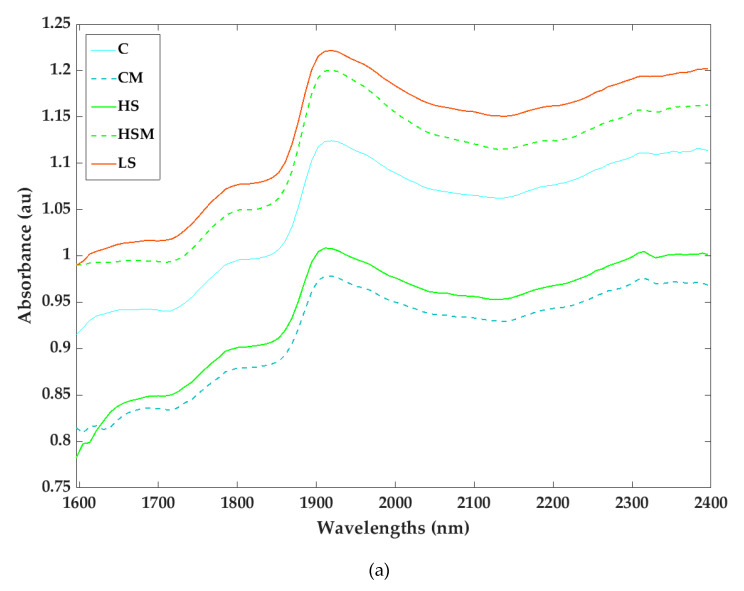

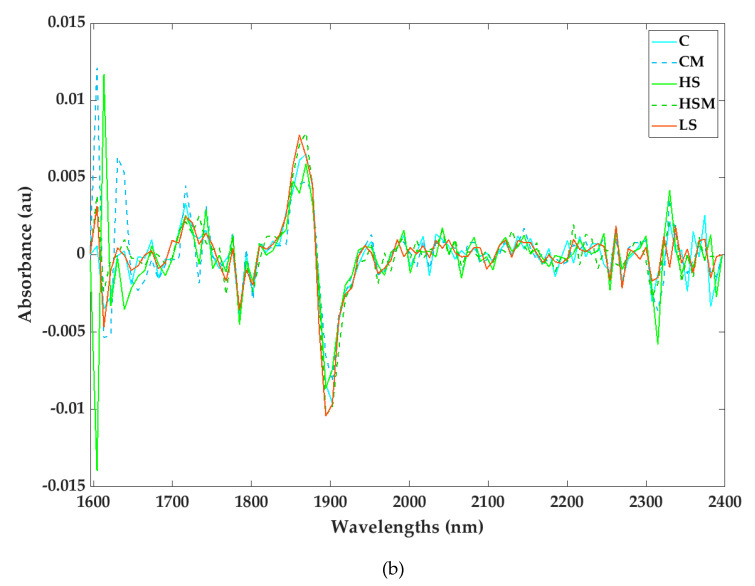
Raw berry absorbance (**a**) and second derivative spectra (**b**) measured with the microPHAZIR^TM^ NIR analyzer for the different smoke and misting treatments. Abbreviations: C = control without misting; CM = control with misting; HS = high density smoke without misting; HSM = high density smoke with misting; and LS = low density smoke.

Absorbance spectra for the averages of replicates for both raw and transformed leaf absorbance spectra are depicted in Figure 3 and Figure 4. For the microPHAZIR^TM^ RX Analyzer leaf absorbances, clear differences in spectral readings were observed for each smoking treatment. A peak was observed at approximately 1784–1793 nm (Figure 3a), while for the transformed data (Figure 3b), large peaks are present between 1596–1647 nm. 

Differences in absorption readings were also found for the microPHAZIRTM RX Analyzer berry absorbance spectra (Figure 4a). Peaks were originally observed at approximately 1785 and 1902 nm, but in the transformed data (Figure 4b), large peaks were observed between approximately 1596–1640 nm and 1820–1940 nm. 

### 3.4. Principal Component Analysis

**Figure 5:**
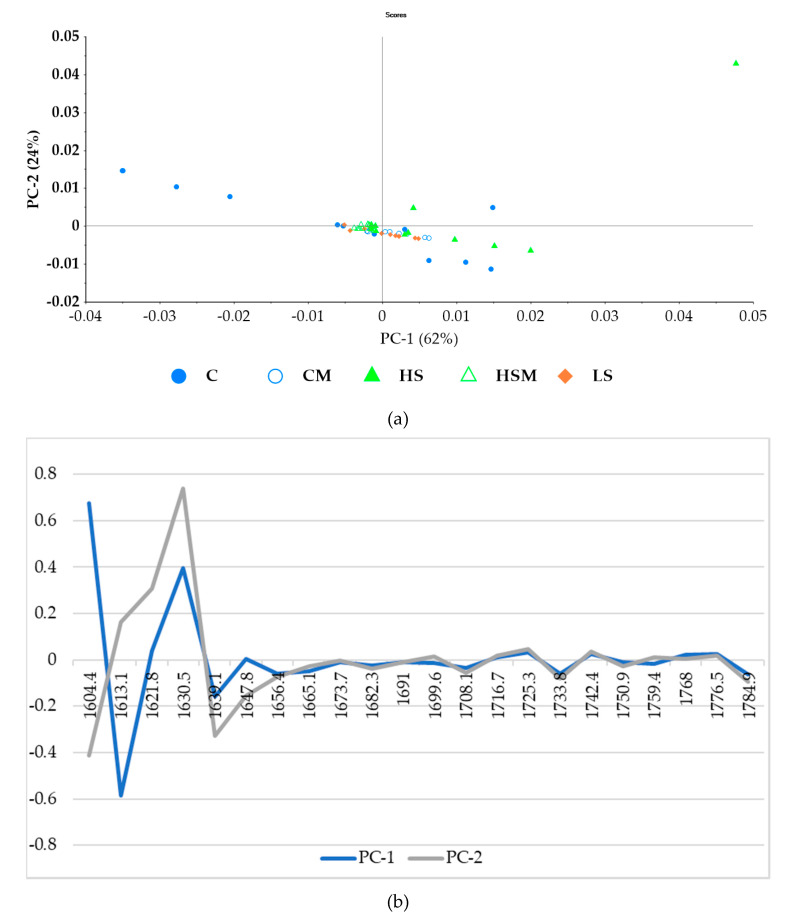
Principal component analysis (PCA) for the microPHAZIR^TM^ leaf absorbance values between 1600–1800 nm (**a**) and loadings (**b**). Abbreviations: C = control without misting; CM = control with misting; HS = high density smoke without misting; HSM = high density smoke with misting; and LS = low density smoke.

Figure 5a shows the principal component analysis (PCA) for the microPHAZIR^TM^ RX Analyzer leaf spectra with absorbance values between 1600–1800 nm. The first principal component (PC1) accounted for 62% of the data variability, while principal component two (PC2) accounted for 24%. Hence, 86% of the total variability was explained by these PCs. There was no clear separation of the different smoke treatments when modeled with the microPHAZIR^TM^ leaf spectra. PC1 was represented by wavelengths between 1604–1621 nm and between 1621–1647 nm (loadings shown in Figure 5b). PC2 was represented by wavelengths between 1613–1647 nm, as well as 1604 nm.

**Figure 6:**
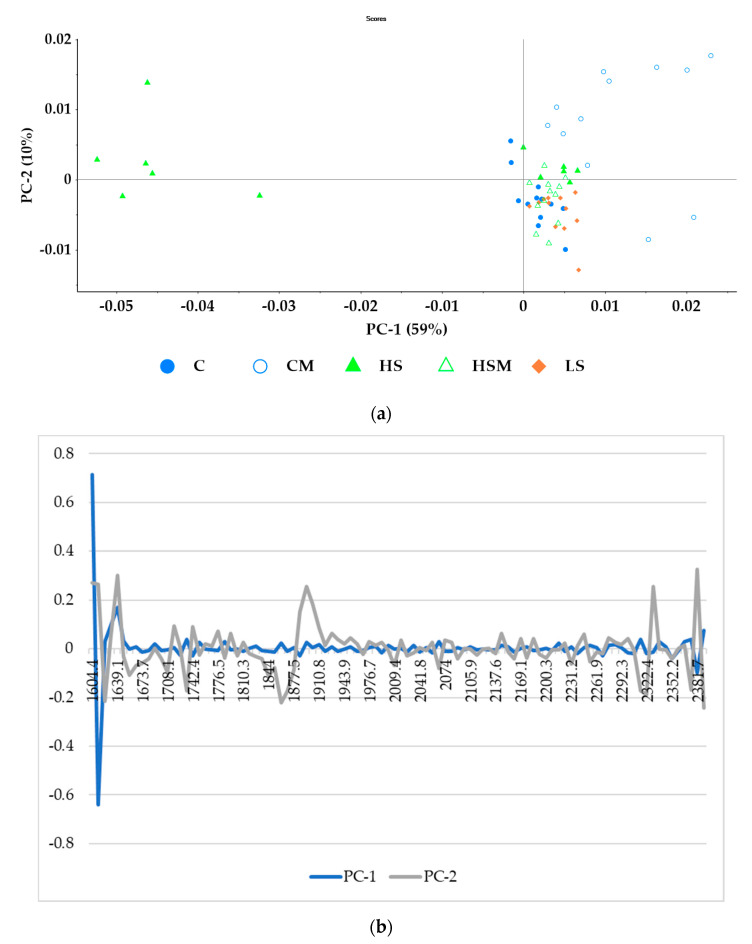
Principal component analysis (PCA) for the microPHAZIR^TM^ berry absorbance values between 1600–2396 nm (**a**) and loadings (**b**). Abbreviations: C = control without misting; CM = control with misting; HS = high density smoke without misting; HSM = high density smoke with misting; and LS = low density smoke.

Figure 6a shows the PCA for the microPHAZIR^TM^ RX Analyzer berry spectra, where 59% of the data variability was described by PC1, while PC2 accounted for 10% of the data variability; thus, a total of 69% of the total data variability was explained by the first two components of the PCA. As with the microPHAZIR^TM^ RX Analyzer leaf spectra, most of the smoke treatments overlapped quadrants. The CM treatment was grouped primarily in the upper right quadrant, while C and LS treatments were grouped primarily in the lower right. The HS treatment was located primarily in the upper right and left quadrants, while the HSM treatment was grouped in the left upper and lower quadrants. PC1 one was represented by the wavelength region 1604–1622. PC2 was represented by the wavelengths between 1630–1647 nm and 2374–2389 nm (loadings shown in Figure 6b).

### 3.5. Spectral Indices

Results for the spectral indices are shown in Table 3. In the case of the leaf NDVI and NAI, the HS and C treatments had the lowest mean values (0.72 and 0.64 for the HS treatment and 0.84 and 0.74 for the C) (*P* < 0.05). There were no differences for the remaining treatments. For the leaf PSRI, the HS treatment had the highest mean value at 0.065, with no differences for the remaining treatments. For the leaf CRI_500_, the LS and HS treatments had the highest values at 1.45 and 1.20, respectively, and for the CRI_700,_ the LS treatments had the highest mean values at 1.76, with no differences for the remaining treatments. 

In the case of the berry NAI, the HS and LS treatments had the highest mean values with 0.88 and 0.87, with both the C and LS treatments having the lowest mean values of 0.80 and 0.75. For the PSRI, both the LS and C treatments had the highest mean values of 0.02, while the HSM had the lowest value at −0.02. 

### 3.6. Artificial Neural Network Models

Table 4 shows the confusion matrices for the two models developed using the spectral readings as inputs and the experimental treatments as targets. Both models displayed high accuracy in classifying the spectral readings according to the treatments, with an overall accuracy of 98% for the microPHAZIR^TM^ leaf model (Model 1) and 97.4% for the microPHAZIR^TM^ berry model (Model 2). Models 1 and 2 presented validation accuracies (94% and 93%, respectively) close to those of the training stage (100% both models). Furthermore, performance values for training (Models 1 and 2: MSE < 0.01) were lower than the other stages and validation (Model 1: MSE = 0.02; Model 2: MSE = 0.03) and testing (Model 1: MSE = 0.02; Model 2: MSE = 0.04) were similar; this indicates that there were no signs of overfitting for both Model 1 and Model 2. 

**Figure 7:**
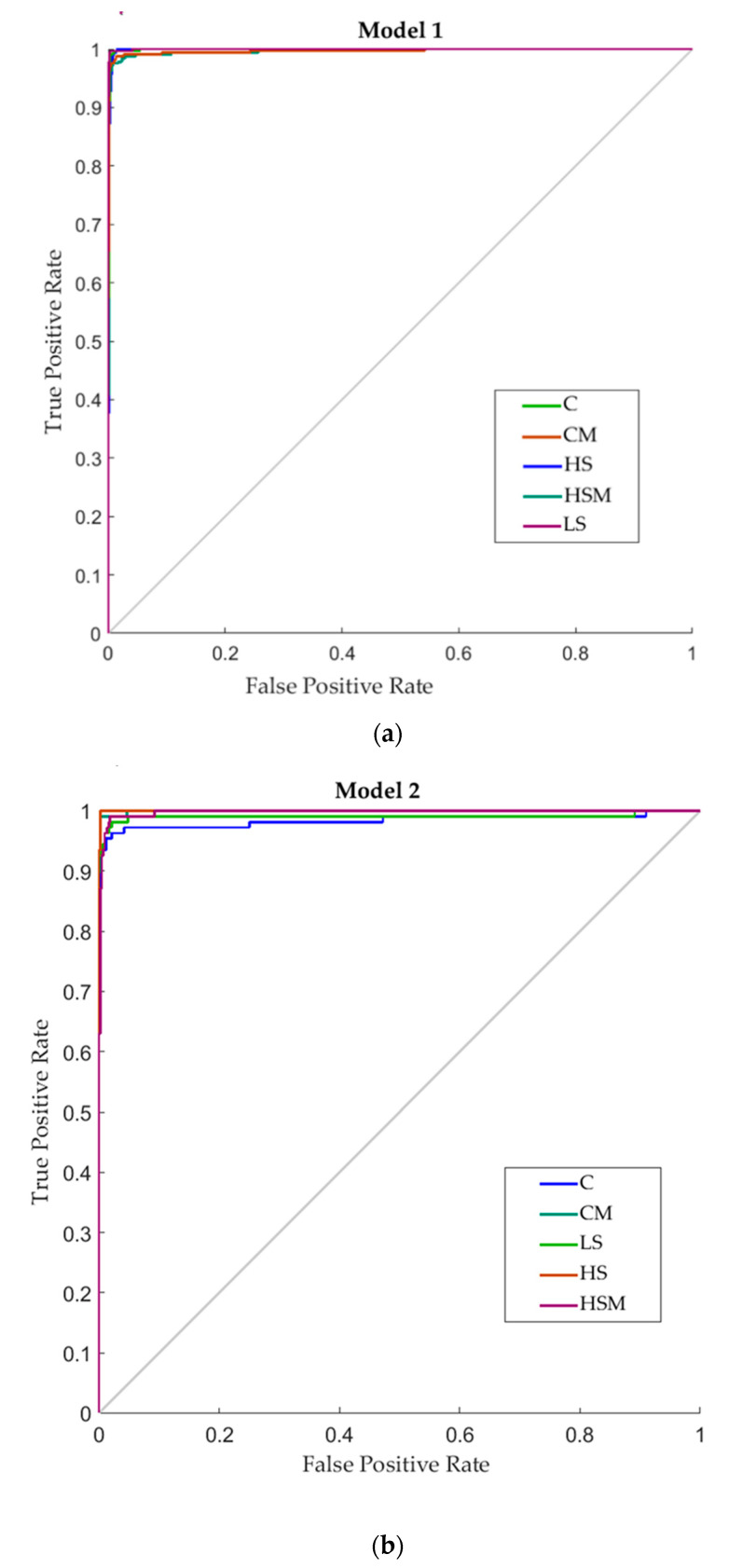
Receiver operating characteristic (ROC) curves for the two models developed (**a**) the microPHAZIR^TM^ leaf model, (**b**) the microPHAZIR^TM^ berry model. Colored lines represent the different smoking treatments. Abbreviations: C = control without misting; CM = control with misting; HS = high density smoke without misting; HSM = high density smoke with misting; and LS = low density smoke.

Figure 7 depicts the receiver operating characteristic (ROC) curves for the two ANN models developed. All models showed high true-positive rates (sensitivity) and low false-positive rates (specificity) for classifying the spectral readings according to the experimental treatment, which can also be observed in the last column of each confusion matrix. For Model 2, the HS treatment had the highest sensitivity (100%), followed by the CM and HSM treatments (99.1% each) and LS treatment (96.3%). The C treatment had the lowest sensitivity of 92.6% for this model. For Model 1, the C treatment had the highest sensitivity (99.1%), followed by the LS treatment (98.8%), HS treatment (97.8%), and CM treatment (97.5%), while the HSM had the lowest sensitivity of 96.9%. 

## 4. Discussion

### 4.1. Physiological Measurements

Leaf gas exchange parameters were measured the day after smoking. The three smoke treatments showed significant reductions in g_s_, in particular, the high-density smoke without misting (HS) treatment, which showed the lowest average reading for g_s_ (Table 2). Stomatal closure is one of the first responses to smoke exposure undertaken by plants [6,26], and a study by Ristic and colleagues [26] found that the time required for g_s_ to recover following one hour of smoke exposure for Cabernet Sauvignon grapevines was approximately 6–10 days. A previous study by Bell et al. [6] found that g_s_ of potted Cabernet Sauvignon grapevines had returned to 60% of pre-smoke exposure rate following fifteen min exposure to smoke using Tasmanian blue gum (*Eucalyptus globulus* L.) leaves as fuel, while rates had returned to 80% of pre-smoke values following exposure to smoke derived from Coast Live Oak (*Quercus agrifolia* Née) leaves. This indicates that in addition to the type of fuel used, the intensity of smoke exposure may also affect the extent of stomatal closure and, hence, reduction in g_s_. It is, therefore, not surprising that the HS treatment had the lowest g_s_. However, it is interesting that the low smoke treatment (LS) had lower g_s_ than the high smoke with misting treatment (HSM), which indicates that misting may have reduced the effect of smoke exposure on g_s_. During a bushfire, the type of fuel burnt will vary depending on the region and the type of plant species native to the area, as well as the amount of smoke exposure due to land topography and wind vectors; therefore, the effect on g_s_ may vary [17,18,23,66]. While misting only partially prevented the uptake of volatile phenols and glycoconjugates in grapes [60], it did appear to have a physiological effect. It is evident that misting reduced the effect of smoke exposure on g_s_. Smoke contains a complex mixture of gases such as sulfur dioxide (SO_2_), O_3,_ and nitrogen dioxide (NO_2_), as well as dust particles that have been shown to inhibit photosynthesis and affect stomatal opening [6,26,29]. Stomata are the primary point of entry for these gases and dust particles [6]; therefore, misting may help prevent the uptake of dust and other particles by trapping them in water that has condensed on the leaf surface, preventing their entrance into the stomata. The present water may also act as a solvent for gases such as SO_2_ and NO_2_, thereby incorporating them into a solution that then may drip off the leaf surface. In addition to this, smoke exposure may trigger stomatal closure by producing high vapor pressure deficits [26,29]. The presence of misting may help reduce the leaf-to-air vapor pressure difference produced by smoke exposure, thereby reducing the impact on g_s_. Misting also appeared to reduce the effect of smoke exposure on transpiration rate (E) as there were no differences between the two control treatments and the LS and HSM treatments. Only the HS treatment had significantly reduced E. Mean rates of photosynthesis (A) followed similar patterns to g_s_, with the HS treatment having the lowest value, followed by the LS treatment and then the HSM treatment, while the control without misting (C) had the highest rate of A. This indicates that while misting may have reduced A in the control treatments, it may also help reduce the effects of smoke exposure on A. 

### 4.2. Near-Infrared Spectroscopy Patterns and Principal Component Analysis

From the PCA biplots (Figure 5 and Figure 6) and spectra (Figure 3 and Figure 4) generated in the current study, it is evident that smoke exposure alters the NIR spectral signals of grapevine leaves and berries, and this may prove useful for the detection of grapevine smoke contamination. For the microPHAZIR^TM^ RX Analyzer leaf spectra, high loadings (Figure 5b.) were observed for the wavelength regions between 1604–1621, 1621–1647, and 1613–1647 nm, all of which correspond to C-H stretching of sugars and aromatic compounds [67,68,69,70]. For the microPHAZIR^TM^ RX Analyzer berry spectra, high loadings (Figure 6b.) were observed for the wavelength regions between 1604–1622, 1630–1647, and 2374–2389 nm, which correspond to C-H stretching of sugars, such as glucose, as well as aromatic hydrocarbons, which may be due to the presence of smoke-derived volatile phenols, such as guaiacols, cresols, and syringols, and their glycoconjugates [67,68,71,72]. 

### 4.3. Spectral Indices

#### 4.3.1. Leaf

The normalized difference vegetation index (NDVI) gives an indication of plant vigor and fruit ripening resulting from relative changes in chlorophyll content. It is based on the variation between the maximum absorption of red by chlorophyll pigments and the maximum reflectance in the infrared caused by leaf cellular structure [56,57,73,74,75]. Similarly, relative changes in anthocyanin content are expressed as the normalized anthocyanin index (NAI). Both the NDVI and NAI are expressed as a normalized value between −1 (lack of green or redness) to +1 (green or red) [56,57]. Not surprisingly, HS leaves had the lowest NDVI and NAI values. Previous studies investigating the effects of pollution on leaf pigments found a decrease in photosynthetic pigments following exposure to pollutants, including sulfur dioxide (SO_2_), carbon dioxide (CO_2_), nitrogen dioxide (NO_2_), and ozone (O_3_) [59,76,77]. These studies are often used as comparisons for investigating the effects of smoke exposure on leaves as compounds in air pollution can also found in smoke [6,22]. There were no differences in NDVI and NAI values between the LS, HSM, and control treatments (C and CM), indicating that misting may reduce the effects of smoke exposure on leaf pigments, and low levels of smoke exposure for one hour may also have no effect. Longer periods of smoke exposure (days or weeks, as is often the case with wildfires) may be required to cause a noticeable change in leaf pigments. 

The plant senescence reflectance index (PSRI) gives an indication of the stage of leaf senescence and fruit ripening through assessing changes in carotenoid accumulation and their proportion to chlorophyll. Values range from −1 to +1, with higher values indicating increased stress and carotenoid accumulation [55,63,64,65,78]. The PSRI was highest for the HS treatment, indicating heightened stress and leaf senescence. This also corresponds with the high CRI_500_ value for this smoke treatment, indicating increased carotenoid accumulation.

#### 4.3.2. Berries

Research by Noestheden et al. [5] found that smoke exposure induced changes in phenylpropanoid metabolites in Pinot Noir berries and wine, some of which are associated with the color and mouthfeel of the wine. Berries exposed to HS and LS treatments had the highest mean NAI values, indicating that smoke exposure may increase anthocyanin content, possibly due to an increase in phenolic accumulation as a stress response induced by exposure to ozone present in smoke [5,79,80]. The HSM treatment had a low NAI value, indicating that misting may reduce anthocyanin concentrations through increased irrigation. Castellarin et al. [81] found that early (before véraison) and late (after the onset of ripening) season, water deficits increased anthocyanin accumulation during ripening. The application of in-canopy misting may reduce water stress and, therefore, reduce anthocyanin accumulation. 

Interestingly the HSM followed by the HS treatments had the lowest PSRI values. As carotenoid concentrations in grapes generally decrease during véraison, this may have resulted in lower PSRI values. Therefore, the PSRI may not be suitable for assessing the degree of ripening in grape berries.

### 4.4. ANN Modeling

Both ANN models classified leaf and berry readings as a function of smoke exposure with high accuracy. The microPHAZIR^TM^ leaf model (model 1) had the highest positive classification, with 98% accuracy (Table 4). The NIR region selected for use in Model 1 was between 1600–1800 nm in order to minimize any possible interference due to the absorption spectra of water in the region of approximately 1930 nm [69]. Furthermore, the region between 1680–1690 nm is associated with aromatic C-H stretching [67]; as such, any patterns observed by the ANN would most likely be due to the presence of smoke-derived volatile phenols. Research by Kennison [22] found a positive correlation between levels of smoke-derived compounds found in leaves and levels in wine; this ANN model developed may, therefore, offer a rapid, in-field method for assessing grapevine smoke contamination. It also demonstrates great promise for further research into the use of NIR spectroscopy coupled with unmanned aerial vehicles (UAVs) with Global Positioning System (GPS) trackers, which could fly over vineyards to scan grapevine canopies and provide maps of smoke contaminated regions. 

The microPHAZIR^TM^ berry model (model 2) also had a high overall accuracy in classifying grape berries according to smoke treatment (97.4%). For Model 2, the entire wavelength range between 1600–2396 nm was used. This includes the C-H stretching of aromatic compounds at 1680 nm, O-H stretching at 1930 nm associated with glucose, cellulose, and water, and C=O second overtone associated with carboxylic acids and water between 1900–1910 nm [67,69]. As NIR measurements were conducted in-field on whole berries, this offers a non-destructive tool for assessing grapevine smoke contamination. Whole grapes may be used for assessment as smoke compounds have been found to occur primarily in grape skins [3,25]. Furthermore, the Lighting Passport^TM^ smart handheld spectrometer may be of interest to growers due to its affordability compared to other spectrometers. It is also very small and lightweight, making it easy to undertake measurements in-field, and it can be connected to smartphones via Bluetooth, where data can be stored and retrieved for later analysis [82]. 

The two ANN models more accurately differentiated the spectral readings relative to PCA. This may be because ANNs are better suited to handle complex, non-linear data, and more readily find patterns or relationships between data than other forms of analysis [53,83,84,85]. Research by Janik et al. [53] found that the combination of ANNs with partial least squares (PLS) or PCA overcomes issues of non-linearity as well as increasing the accuracy of regression models in predicting total anthocyanin concentrations in red grape homogenates. This may also explain why Model 2 was able to accurately differentiate the berry spectral readings from C, CM, and LS treatments, despite analysis of variance indicating there were no statistically significant differences.

As smoke exposure altered the chemical fingerprinting of grapevine leaves and berries, the ANN models were able to detect changes in the spectral patterns and then classify the readings as a function of experimental treatments. This may offer grape growers a rapid method of assessing the level of smoke contamination in grape berries and leaves, with a high level of accuracy and precision. This may assist growers in deciding which berry samples to send for further chemical analysis to quantify the levels of smoke compounds in grapes and predict the level of smoke taint in the final wine, or they may decide to avoid harvesting heavily contaminated grapes for winemaking. Furthermore, as this method is non-destructive, repeated measurements are possible. By knowing the level of smoke contamination, growers can make informed decisions.

While the ANN models developed were able to classify Cabernet Sauvignon leaf and berry spectra accurately, further research is required to assess whether these models can be used for other grape varieties, as differences in berry composition and leaf physiology may affect the accuracy of classification [6,34]. Previous research evaluated MIR spectroscopy for the classification of smoke tainted wines found compositional differences due to grape variety prevailed over differences resulting from low levels of smoke exposure [34]. Furthermore, the physiological responses of different grape varieties to smoke were found to vary, both in magnitude and in recovery time [6,26]. Thus, further testing of these models using berry and leaf spectra from different grapevine varieties is required.

## 5. Conclusions

Results from this study indicate that smoke exposure alters the NIR spectra of Cabernet Sauvignon grapevine leaves and berries. As a result, accurate classification models can be developed using ANN modeling. Artificial neural networks are better at classifying non-linear or complex data than traditional techniques, such as principal component analysis. Furthermore, the use of UV-Vis spectroscopy may offer insights into the physiological performance of leaves and the quality and degree of ripening of grapes. These techniques may assist grape growers in identifying grapevines that have been contaminated by smoke, thereby informing decision-making to avoid harvesting and processing heavily contaminated grapes and/or the need for mitigation techniques to manage the risk of smoke taint in resulting wine. Further testing of the ANN models developed in the current study is required to assess their accuracy in classifying grapevine leaf and berry spectra from other grape varieties.

## Figures and Tables

**Table 1:** Calculations for the spectral indices investigated in this study.

Index Name	Index Abbreviation	Equation	References
Normalized difference vegetation index	NDVI	$\frac{\left(I780-I660\right)}{\left(I780+I660\right)}$	[56,57]
Normalized anthocyanin index	NAI	$\frac{\left(I780-I570\right)}{\left(I780+I570\right)}$	[56,57]
Carotenoid reflectance index	CRI_550_	$\frac{1}{I510}-\frac{1}{I550}$	[63,64]
Carotenoid reflectance index	CRI_700_	$\frac{1}{I510}-\frac{1}{I700}$	[65]
Plant senescence reflectance index	PSRI	$\frac{I680-I500}{I750}$	[59]

**Table 2:** Gas exchange parameters measured for the different smoke treatments.

Smoke Treatment	E (mmol m^−2^ s^−1^)		g_s_ (mol m^−2^ s^−1^)		A (µmol m^−2^ s^−1^)	
	Mean	SD	Mean	SD	Mean	SD
C	2.48 ^a^	0.70	0.15 ^a^	0.05	10.77 ^a^	3.46
CM	2.31 ^a^	0.54	0.15 ^a^	0.05	9.66 ^ab^	2.31
HS	1.43 ^b^	0.62	0.06 ^c^	0.03	5.59 ^d^	2.8
HSM	2.06 ^a^	0.44	0.10 ^b^	0.03	8.15 ^bc^	1.97
LS	2.18 ^a^	0.78	0.08 ^bc^	0.03	7.01 ^cd^	2.42

Abbreviations: C = control without misting; CM = control with misting; HS = high density smoke without misting; HSM = high density smoke with misting; and LS = low density smoke; SD = standard deviation. Means followed by different letters are significantly different based on Fisher least significant difference (LSD) post hoc test (*α* = 0.05).

**Table 3:** Means and standard deviation (SD) of spectral indices calculated for leaves and berries.

Treatment	Leaf										Berry			
	NDVI		NAI		PSRI		CRI500		CRI700		NAI		PSRI	
	Mean	SD	Mean	SD	Mean	SD	Mean	SD	Mean	SD	Mean	SD	Mean	SD
CM	0.85 ^a^	0.10	0.77 ^a^	0.11	0.00 ^b^	0.01	0.70 ^b^	0.64	0.82 ^b^	0.79	-	-	-	-
C	0.84 ^ab^	0.08^2^	0.74 ^ab^	0.11	0.01 ^b^	0.02	0.67 ^b^	0.78	0.77 ^b^	0.87	0.80 ^b^	0.07	0.02 ^a^	0.02
HS	0.72 ^b^	0.50	0.64 ^b^	0.49	0.07 ^a^	0.19	1.20 ^a^	0.24	0.82 ^b^	0.62	0.88 ^a^	0.04	0.00 ^b^	0.00
HSM	0.87 ^a^	0.11	0.79 ^a^	0.11	0.00 ^b^	0.02	0.48 ^b^	0.06	0.58 ^b^	0.45	0.75 ^b^	0.10	−0.02 ^c^	0.00
LS	0.92 ^a^	0.04	0.84 ^a^	0.08	0.00 ^b^	0.01	1.45 ^a^	1.08	1.76 ^a^	1.40	0.87 ^a^	0.05	0.02 ^a^	0.01

Abbreviations: C = control without misting; CM = control with misting; HS = high density smoke without misting; HSM = high density smoke with misting; and LS = low density smoke. Means followed by different letters are statistically significant based on Fisher’s least significant difference (LSD) post hoc test (*α* = 0.05).

**Table 4:** Statistical results for the artificial neural networks pattern recognition models. Model 1: microPHAZIRTM for leaves, and Model 2: microPHAZIRTM for berries. Performance is based on means squared error (MSE).

Stage	Samples (n)	Accuracy %	Error %	Performance (MSE)
Model 1				
Training	1131	100	0	0.00
Validation	243	94.2	5.8	0.02
Testing	243	92.6	7.4	0.02
Overall	1617	98.0	2	-
Model 2				
Training	378	100	0	0.00
Validation	81	92.6	7.4	0.03
Testing	81	90.1	9.9	0.04
Overall	540	97.4	2.6	-

## Supplementary Materials

The following are available online at https://www.mdpi.com/1424-8220/20/18/5099/s1, Table S1: Concentrations of volatile phenols in grape juice (µg/L) and their glycoconjugates in grape homogenate (µg/kg) one hour after smoke treatments.
