# Supplementary material for: Classification of Smoke Contaminated Cabernet Sauvignon Berries and Leaves Based on Chemical Fingerprinting and Machine Learning Algorithms
Source: Sensors (Basel). 2020 Sep 7;20(18):5099. doi: 10.3390/s20185099 (PMC7571113; doi:10.3390/s20185099)
Supplement: Supplementary file 1 [file sensors-20-05099-s001.pdf]

Supplementary material

Table S1. Concentrations of volatile phenols in grape juice (μg/L) and their glycoconjugates in grape homogenate (μg/kg) one hour after smoke treatments

	Smoke compound	C		CM		HS		HSM		LS	
		Mean	SD	Mean	SD	Mean	SD	Mean	SD	Mean	SD
Volatile phenols	guaiacol	10[a]	6	3[a]	0	108[b]	34	76[c]	5	12[a]	2
	4-methylguaiacol	4[a]	1	4[a]	0	20[b]	8	13[b]	2	4[a]	0
	phenol	3[a]	2	2[a]	0	55[b]	32	39[b]	8	7[a]	2
	4-methylsyringol	3[ab]	1	2[b]	0	17[c]	7	9[a]	1	3[b]	0
	syringol	21[ab]	7	8[a]	3	126[c]	56	59[b]	5	25[ab]	6
	total cresols	5[a]	3	3[a]	1	83[b]	33	59[b]	10	12[a]	1
Volatile phenol glycoconjugates	GuPG	5.0[a]	0.7	2.7[a]	0.5	18.7[b]	6.1	7.6[a]	0.5	4.0[a]	0.8
	GuGG	0.1[a]	0.0	0.1[a]	0.0	1.1[b]	0.3	0.6[c]	0.0	0.2[a]	0.0
	GuMG	0.4[a]	0.4	0.1[a]	0.1	24.4[b]	7.3	9.0[c]	2.1	1.5[a]	0.2
	GuRG	nd	-	nd	-	1.0[a]	0.3	0.7[a]	0.2	1.0[a]	0.3
	MGuPG	1.2[a]	0.5	0.6[a]	0.1	9.2[b]	2.2	4.5[c]	0.5	1.0[a]	0.1
	MGuRG	0.9[a]	0.4	0.8[a]	0.2	4.5[b]	0.9	2.3[c]	0.2	1.0[a]	0.1
	PhRG	0.6[a]	0.2	0.4[a]	0.1	3.0[c]	0.8	1.3[b]	0.1	1.0[ab]	0.1
	PhGG	0.0[a]	0.0	0.0[a]	0.0	0.4[c]	0.1	0.2[b]	0.0	0.1[ab]	0.0
	PhPG	3.0[a]	1.4	1.6[a]	0.2	17.8[c]	6.2	8.7[b]	0.4	4.0[ab]	0.4
	PhMG	0.2[a]	0.1	0.1[a]	0.1	1.0[b]	0.3	1.6[b]	0.9	0.2[a]	0.0
	CrPG	11.8[a]	3.2	8.4[a]	0.3	35.7[c]	10.0	0.5[b]	0.0	13.3[ab]	0.7
	CrGG	0.6[a]	0.1	0.5[b]	0.1	0.5[ab]	0.0	0.5[b]	0.0	0.2[c]	0.1
	CrRG	2.7[a]	0.8	1.8[a]	0.2	10.9[b]	2.7	5.9[c]	0.3	3.2[a]	0.4
	SyGG	3.7[a]	1.3	2.5[a]	0.2	44.4[c]	11.1	17.5[b]	2.0	9.1[ab]	0.7
	SyMG	1.1[a]	0.8	0.3[a]	0.1	22.8[b]	5.7	6.6[c]	0.8	1.3[a]	0.1
	SyPG	1.1[a]	0.3	0.9[a]	0.1	3.6[c]	0.8	2.0[b]	0.1	1.4[ab]	0.1
	MSyGG	1.0[a]	0.6	0.4[a]	0.0	10.7[b]	2.3	4.0[c]	1.1	1.5[a]	0.1
	MSyPG	0.1[a]	0.0	0.1[a]	0.0	0.4[b]	0.0	0.2[c]	0.1	0.1[a]	0.0

Abbreviations: C = control without misting; CM = control with misting; LS = low density smoke exposure; HS = high density smoke exposure without misting; HSM = high density smoke exposure with misting. Gu = guaiacol; Cr = cresol; Ph = phenol; Sy = syringol; 4MG = 4-methylguaiacol; MSy = 4-methylsyringol; MG = monoglucoside; GG = glucose-glucoside; PG = pentose-glucoside; R = rutinoside; SD= standard deviation; nd = not detected. Means followed by different letters are statistically significant based on Fisher's least significant difference (LSD) *post hoc* test (α= 0.05).
